# Inhibition of the mTORC1/NF-*κ*B Axis Alters Amino Acid Metabolism in Human Hepatocytes

**DOI:** 10.1155/2021/8621464

**Published:** 2021-01-18

**Authors:** Meng Zhang, Yuting Fu, Yuhao Chen, Yuze Ma, Zhixin Guo, Yanfeng Wang, Huifang Hao, Quan Fu, Zhigang Wang

**Affiliations:** ^1^State Key Laboratory of Reproductive Regulation & Breeding of Grassland Livestock, School of Life Sciences, Inner Mongolia University, Hohhot 010070, China; ^2^School of Life Sciences, Jining Normal University, Jining 012000, China; ^3^School of Fisheries and Life Science, Dalian Ocean University, Dalian 116023, China; ^4^Department of Clinical Laboratory, Affiliated Hospital of Inner Mongolia Medical University, Hohhot 010050, China

## Abstract

In addition to serving as the building blocks for protein synthesis, amino acids can be used as an energy source, through catabolism. The transamination, oxidative deamination, and decarboxylation processes that occur during amino acid catabolism are catalyzed by specific enzymes, including aspartate aminotransferase (AST), glutamate dehydrogenase (GDH), glutamic acid decarboxylase (GAD), and ornithine decarboxylase (ODC); however, the overall molecular mechanisms through which amino acid catabolism occurs remain largely unknown. To examine the role of mechanistic target of rapamycin complex 1 (mTORC1) on amino acid catabolism, mTORC1 was inactivated by rapamycin or shRNA targeting *Raptor*, versus activated by overexpressing *Rheb* or amino acids in human hepatocytes. The expression of amino acid catabolic genes and related transcription factor was investigated by RT/real-time PCR and western blot analysis. A few types of amino acid metabolite were examined by ELISA and HPLC analysis. The data showed that inactivated mTORC1 resulted in inhibition of NF-*κ*B and the expression of *AST*, *GDH*, *GAD*, and *ODC*, whereas activated mTORC1 enhanced NF-*κ*B activation and the expression levels of the catabolism-associated genes. Further, inhibition of NF-*κ*B reduced the expression levels of *AST*, *GDH*, *GAD*, and *ODC*. mTORC1 upregulated NF-*κ*B activation and the expression of *AST* and *ODC* in response to glutamate and ornithine treatments, whereas rapamycin inhibited the utilization of glutamate and ornithine in hepatocytes. Taken together, these results indicated that the mTORC1/NF-*κ*B axis modulates the rate of amino acid catabolism by regulating the expression of key catabolic enzymes in hepatocytes.

## 1. Introduction

The availability of nutrients and energy is critical for cellular growth and proliferation. To achieve cellular homeostasis, cells must efficiently utilize available extracellular and intracellular nutrients, to provide energy when nutrients are scarce [1]. Amino acids can be used to provide energy and energy precursors, through catabolism. The liver is a vital organ in which amino acid catabolism occurs. The primary amino acid catabolic pathways include transamination, oxidative deamination, and decarboxylation, which are catalyzed by specific enzymes, including aspartate aminotransferase (AST), glutamate dehydrogenase (GDH), glutamic acid decarboxylase (GAD), and ornithine decarboxylase (ODC), in liver cells. Serum AST is a clinical marker that is often used for the diagnosis of liver diseases and is commonly used as a marker of severe cellular damage in the liver [2–4]. Glutamate is a nonessential amino acid at the crossroads of nitrogen and energy metabolism and is found at the highest levels in its free form in the liver. Glutamate can be converted into *α*-ketoglutarate via oxidative deamination, which is catalyzed by GDH [5]. GDH plays a major role in amino acid catabolism and ammonia detoxification in the liver. The liver is rich in GDH, which catalyzes the reversible oxidative deamination of glutamate into *α*-ketoglutarate and ammonia, bridging the amino acid-to-glucose pathway [6–8]. Moreover, glutamate can also be converted into *γ*-aminobutyric acid (GABA), via decarboxylation by GAD, which protects hepatocytes from ethanol cytotoxicity, *in vitro* [9]. Ornithine, a nonproteinogenic amino acid that is not utilized during *de novo* protein synthesis, is derived from arginine, via citrulline, and the decarboxylation of ornithine by ODC results in the generation of putrescine in mammalian cells [10, 11]. A sharp increase in polyamine (putrescine, spermidine, and spermine) synthesis may favor the rapid proliferation of preneoplastic and neoplastic liver cells. The progressive upregulation of the *ODC* expression and increases in ODC activity and polyamine synthesis occurs during rat hepatocarcinogenesis [12]. Amino acid catabolism enzymes have been associated with liver diseases.

Mechanistic (formerly mammalian) target of rapamycin (mTOR) complex 1 (mTORC1) is a nutrient-sensitive, multiprotein complex that links nutrient and energy signals and functions as a master regulator of cell growth and metabolism during specific energy- and nutrient-consuming processes [13–15]. Amino acid availability serves as a signal that can initiate mTORC1 signaling, recruiting mTORC1 to the lysosomal surface, where it is activated by the small GTPases Rheb and Rags [16–19]. mTORC1 has been demonstrated to use distinct mechanisms to sense several types of amino acids in the lysosome and cytosol [20–23]. However, the function of mTORC1 during amino acid catabolism remains poorly understood.

Rapamycin treatment can reduce the plasma levels of alanine aminotransferase (ALT) and AST in cirrhotic rats [24]. The inhibition of mTOR activity by rapamycin and the reduction of ribosomal protein S6 expression using small interfering RNA (siRNA) inhibited GDH and GLS activity in ovarian cancer cells [25]. ODC mRNA was shown to be stabilized in an mTORC1-dependent manner in Ras-transformed rat intestinal epithelial (RIE-1) cells [26]. At present, the relationship between GAD and mTOR signaling is unclear.

The transcription factor nuclear factor (NF-) *κ*B is the best known as a central regulator of inflammation and has recently attracted attention for its involvement in metabolic disorders [27–29]. Cooperative NF-*κ*B/STAT (signal transducer and activator of transcription) signaling regulates metabolic reprogramming and aspartate transaminase (GOT2) gene expression in lymphoma cells [30]. Several lines of evidence support a crucial role for NF-*κ*B in the governance of energy homeostasis and the mediation of metabolic reprogramming in cancer cells [31]. Thus, NF-*κ*B is thought to play a role in cell metabolism.

Intracellular amino acids play multiple functions in protein synthesis and energy homeostasis, and these functions are related to mTORC1 [13–15]. However, compared with the regulation of protein synthesis, little is known regarding the function of mTORC1 in the regulation of amino acid catabolism processes. We previously demonstrated that mTORC1 regulates peptidoglycan-induced inflammation, via NF-*κ*B signaling, in murine macrophages [32]. Furthermore, other reports have indicated that NF-*κ*B is involved in energy homeostasis and metabolic reprogramming [30, 31]. Therefore, we hypothesized that mTORC1 regulates amino acid catabolic gene expression via NF-*κ*B signaling, to modulate amino acid catabolism in hepatocytes. The purpose of this study was to determine the functions and mechanisms of the mTORC1/NF-*κ*B axis on amino acid catabolism by measuring the expression of *AST*, *GDH*, *GAD*, and *ODC*. We also investigated whether mTORC1 regulates the expression of *AST* and *ODC* in response to changes in glutamate or ornithine levels and whether rapamycin inhibits the utilization of glutamate and ornithine in hepatocytes. The results of this study provide insights into the precise mechanism by which amino acid catabolism is regulated in human hepatocytes.

## 2. Materials and Methods

### 2.1. Cell Lines and Culture Conditions

HL-7702 hepatocytes were maintained in 1640 medium, supplemented with 10% heat-inactivated fetal bovine serum. All cells were cultured in humidified air, with 5% CO_2_ at 37°C, as described in our previously reported methods [33]. Cells were thawed, and P2–P10 cells were used in all experiments.

### 2.2. Reagents and Antibodies

Rapamycin (Gene Operation, Ann Arbor, MI, USA) was dissolved in ethanol (Sigma-Aldrich, Inc., USA), to a stock concentration of 50 mg/mL, and stored at -20°C. Rapamycin was diluted with culture medium to the final target concentration of 100 nM before use. SC75741 (America Selleck Biotechnology Co. Ltd., Houston, Texas, USA) was dissolved in dimethyl sulfoxide (DMSO), to a stock concentration of 50 mM, and was diluted to the final target concentration of 10 *μ*M with culture medium before use. The concentration of ethanol in the final solution did not exceed 0.5% (*v*/*v*) in any experiment. The following primary antibodies were purchased from Cell Signaling Technology, Inc. (Beverley, MA, USA): anti-NF-*κ*B p65, anti-phospho-NF-*κ*B p65 (Ser536), anti-p-S6 (Ser240/244), and anti-Raptor. Anti-S6 primary antibody was purchased from Santa Cruz Biotechnology, Inc. (CA, USA). Anti-p-mTOR (Ser2448) and anti-mTOR antibodies were purchased from Abcam (plc 330 Cambridge Science Park, Cambridge, UK). Anti-*β*-actin primary antibody was purchased from Sigma-Aldrich, Inc. (St. Louis, MO, USA). Enhanced chemiluminescence (ECL) anti-rabbit IgG-horseradish peroxidase (HRP) and ECL anti-mouse IgG-HRP were obtained from GE Healthcare (Little Chalfont, Buckinghamshire, UK).

### 2.3. Enzyme-Linked Immunosorbent Assay (ELISA)

Hepatocytes were seeded in 6-well plates, at 1 × 10^6^ cells per well, and cultured until they reached 80% confluence. To determine the intracellular concentrations of glutamate, oxaloacetate, *α*-ketoglutaric acid, and aspartic acid, hepatocytes were cultured in serum-free medium for 13 hours, followed by amino acid starvation for 1 hour, and then incubated in the presence of glutamate for 1 hour. During serum starvation, cells were pretreated with 100 nM rapamycin, for 8 hours, or with 10 *μ*M SC75741, for 12 h. The inhibitors were added at the right time during the serum-free medium. To determine the intracellular concentrations of glutamate, oxaloacetate, *α*-ketoglutaric acid, and aspartic acid, three groups were established: control, glutamate, and glutamate with rapamycin. To determine the intracellular concentrations of AST, GDH, ODC, and GAD, hepatocytes were treated with rapamycin or SC75741 or were transfected with pRNAT-U6.1/Neo-shRaptor or pIRES2-EGFP-Rheb. Treated hepatocytes were harvested with trypsin and centrifuged, to remove cell culture supernatants. Cell lysates were prepared by performing 5 freeze-thaw cycles and standardized to the same protein concentrations by adjusting the volumes of the protein lysates. Equal volumes of protein lysates were measured using specific enzyme-linked immunosorbent assay (ELISA) kits (Wuhan Xinqidi Biological Technology Co. Ltd. Wuhan, China), according to the manufacturer's instructions, to measure the levels of glutamate, oxaloacetate, *α*-ketoglutaric acid, aspartic acid, AST, GDH, ODC, and GAD. Absorbance was measured at 450 nm and 630 nm on a Varioskan Flash Multimode Reader (Thermo Fisher Scientific, Pittsburgh, PA, USA). The absorbance values were measured three times in each sample, and the mean value of 3 independent measurements was used during statistical analyses.

### 2.4. Western Blot Analysis

Western blot analysis was used to detect the expression levels of the indicated proteins and phosphorylated proteins, as previously described [32]. Briefly, cells were harvested with trypsin, washed with cold phosphate-buffered saline, and lysed in cell lysis buffer. The cells were then placed on ice for 10 min and centrifuged at 10,625 × g, at 4°C for 10 min. Lysate protein concentrations were determined using the Bradford assay (Bio-Rad Laboratories, USA). Equal amounts (40 *μ*g) of protein were subjected to sodium dodecyl sulfate-polyacrylamide gel electrophoresis (SDS-PAGE; 10% gels (*w*/*v*)), transferred to polyvinylidene fluoride (PVDF) membranes, and incubated with the indicated primary antibody, overnight at 4°C. Membranes were then incubated with HRP-conjugated secondary antibody for 1 hour, at room temperature. ECL reagent (Amersham) was used with the Western Blotting System (GE Healthcare Bio-Sciences, Pittsburgh, PA, USA) to detect proteins of interest. Protein bands were quantified on a Gel-Pro Analyzer 4.0 (Media Cybernetics, USA). The experiment was conducted in triplicate.

### 2.5. RT-qPCR Analysis

Reverse transcription-quantitative real-time polymerase chain reaction (RT-qPCR) was used to determine the mRNA levels of *AST*, *GDH*, *ODC*, *GAD*, *Raptor*, and *Rheb* in hepatocytes, for comparisons between the treatment and control groups. Total RNA from untreated and treated cells was reverse-transcribed with an oligo (dT)_12–18_ primer, using the AMV 1st Strand cDNA Synthesis Kit (Takara Co. Ltd., China). cDNA sequences were amplified using the primers shown in Table S1. The reactions were performed using the KAPA SYBP® FAST qPCR Kit, optimized for the LightCycler® 480 (KAPA BIOSYSTEMS, Inc., Boston, Massachusetts, USA), according to the manufacturer's instructions. One microliter of cDNA was amplified in a 25 *μ*L reaction that contained 10 *μ*M forward primer (0.5 *μ*L), 10 *μ*M reverse primer (0.5 *μ*L), SYBR Premix Ex Taq (12.5 *μ*L), and nuclease-free water (10.5 *μ*L). Cycling conditions consisted of an initial denaturation step at 95°C for 5 min, then 40 cycles at 95°C for 5 sec, 54°C for 30 sec, and 72°C for 20 sec, followed by a final extension at 72°C for 10 min. Three technical replicates were performed for each sample. Expression levels were determined by calculating the 2^-*ΔΔ*CT^ values, and the RT-qPCR results were analyzed by Student's *t*-test, to compare expression levels between untreated and treated groups. Three independent experiments were performed.

### 2.6. DNA Constructs

A short hairpin RNA- (shRNA-) based *Raptor* RNA-silencing construct (shRaptor), with the sequence 5′-aaGCTCTGCACGTCCTTACGTTTCAAGAGAACGTAAGGACGTGCAGAGCtt-3′, was designed and synthesized to construct the pRNAT-U6.1/Neo-shRaptor expression vector. *Rheb* cDNA was amplified, using the forward primer 5′-GTTGGTTGGGAATAAGAAAGAC-3′ and the reverse primer 5′-CACATCACCGAGCATGAAGACT-3′, which were based on the human *Rheb* sequence (GenBank Accession number NM_005614). The *Rheb* PCR fragment was inserted into the pIRES2-EGFP vector (Clontech Laboratories, Inc., Mountain View, CA, USA), to construct the pIRES2-EGFP-Rheb expression vector.

### 2.7. In Vitro Transfection

The plasmids pRNAT-U6.1/Neo-shRaptor and pIRES2-EGFP-Rheb were transfected into HL-7702 cells, using Lipofectamine 2000 (Invitrogen, Carlsbad, New Mexico, USA), according to the manufacturer's instructions. Transfectants were selected by culturing cells in the presence of G418 (Hyclone Laboratories, Inc. Logan, Utah, USA), for 48 hours, and were imaged using a ZEISS AX10 fluorescence microscope (Carl Zeiss Microscopy, Thornwood, NY, USA), before the cells were collected. For the ELISA assay, cell lysates were prepared by 5 freeze-thaw cycles. For western blot analysis, cell lysates were prepared by lysing cell lysis buffer.

### 2.8. HPLC Analysis

High-performance liquid chromatography (HPLC) was performed using an Agilent 1260 liquid chromatography system (Agilent Technologies Inc., Santa Clara, CA, USA) and diode array detector (DAD). HL-7702 cells were cultured with serum-free medium for 13 hours, followed by amino acid starvation for 1 hour, and then incubation with ornithine for 1 hour. During serum starvation, cells were pretreated with 100 nM rapamycin, for 8 hours. Three groups were established: control, ornithine, and ornithine with rapamycin. Cells were collected and dissolved in 1 mL lysis buffer, and protein concentrations were determined.

For the analysis of putrescine, the protein samples were treated with n-hexane to remove lipids, and the mixture was extracted with N-butanol/trichloromethane (1 : 1 *v*/*v* ratio). The extracting agent was removed by aspiration and evaporation to dryness, under a stream of nitrogen, at 40°C. Samples were then dissolved in 0.1 mM HCl for derivatization. For derivatization, the mixture was combined with dansyl chloride, which was then aspirated, and the sample was evaporated to dryness under a stream of nitrogen, at 40°C. Samples were then dissolved in 1 mL methanol for HPLC analysis, using a C18 column (150 mm × 4.6 mm, 5 *μ*m), at 30°C. The mobile phase contained A (methanol) and B (water), which was used according to the following program: 0 min, 55% A; 7 min, 65% A; 14 min, 70% A; 20 min, 70% A; 27 min, 90% A; and 30 min, 100% A. The flow rate was 1.5 mL/min, and the injection volume was 20 *μ*L. Putrescine was tentatively identified by comparing its retention time with that of authentic standards, under identical analysis conditions, at 254 nm.

For the detection of ornithine, 200 *μ*L of each protein sample was mixed with 10 *μ*L 1.0 mg/mL norleucine, 100 *μ*L 1 mM triethylamine-acetonitrile solution, and 100 *μ*L 0.1 mM phenyl isothiocyanate-acetonitrile solution and allowed to stand at room temperature for 1 hour. Next, 400 *μ*L n-hexane was added, and the mixture was allowed to stand for 10 min. The lower clear solution was passed through a 0.45 *μ*m filter. Next, 2.0 *μ*L of each sample was injected into the HPLC system with a DIONEX Acclaim 120 C18 column (250 mm × 4.6 mm, 5 *μ*m) at 40°C (Thermo Fisher Scientific Inc., Waltham, MA, USA). The mobile phase contained A (0.2 mM sodium acetate:acetonitrile solution, *v*/*v* = 93 : 7) and B (water:acetonitrile solution, *v*/*v* = 20 : 80), per the following program: 0 min, 0% B; 5 min, 3% B; 14 min, 11% B; 17 min, 21% B; 29 min, 34% B; and 41 min, 100% B. The flow rate was 1.0 mL/min. Ornithine was tentatively identified by comparing its retention time with that of authentic standards, under identical analysis conditions, at 254 nm. Two independent experiments were performed.

### 2.9. Statistical Analyses

Statistical analyses were conducted using SPSS PASW Statistics for Windows, v18.0 (SPSS Inc., Chicago, IL, USA). Data were analyzed using standard parametric statistics and one-way analysis of variance (ANOVA), followed by Tukey's post hoc comparisons. Data are expressed as the means ± SD. The results are presented as the average of at least three independent experiments unless stated otherwise. Statistical significance was accepted when *p* ≤ 0.05.

## 3. Results

### 3.1. Inactive mTORC1 Downregulates NF-*κ*B Activation and the Expression of Amino Acid Catabolic Genes in Hepatocytes

**Figure 1:**
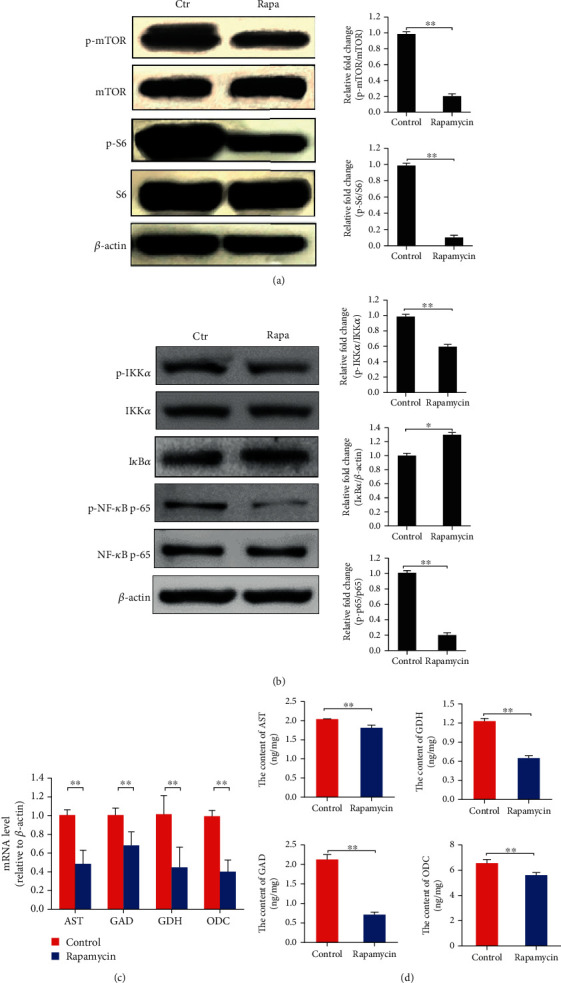
Rapamycin inhibits mTORC1 signaling and NF-*κ*B activation and attenuates the expression of *AST*, *GDH*, *GAD*, and *ODC* in hepatocytes. (a) The phosphorylation of mTOR and S6 was decreased by rapamycin treatment. (b) The phosphorylation of IKK*α* and NF-*κ*B was decreased by rapamycin treatment, and the degradation of I*κ*B*α* was attenuated. (c) The relative mRNA levels of *AST*, *GDH*, *GAD*, and *ODC* were decreased by rapamycin treatment. (d) The intracellular contents of AST, GDH, GAD, and ODC were decreased by rapamycin treatment. Protein bands were quantified using Gel-Pro Analyzer 4.0 (Media Cybernetics, Inc., Rockville, MD, USA) (the values represent the means ± SD, *n* = 3, ^∗^*p* < 0.05, ^∗∗^*p* < 0.01).

Several lines of evidence support a crucial role for NF-*κ*B in metabolic disorders and the mediation of metabolic reprogramming [27, 29, 31]. Previous studies have shown that IKKs are NF-*κ*B subunit p65 kinases, and the phosphorylation site is Ser536 in the COOH-terminal transactivation domain [34]. Inhibition of mTORC1 decreased the level of phosphorylated IKK*α*/*β*, thus reducing the phosphorylation and transcriptional activity of NF-*κ*B [35], and prevented flagellin-induced I*κ*B*α* degradation [36]. To verify NF-*κ*B directs the expression of amino acid catabolism-associated genes, we predicted the transcription factor binding sites (TFBS) and the transcription factor binding motifs (TFBM) of NF-*κ*B by bioinformatics analysis, and the TFBS and TFBM of NF-*κ*B in the promoter sequence of the *AST*, *GDH*, *GAD*, and *ODC* were found (Figure S3, Figure S4). Thus, we hypothesized that mTORC1 regulates amino acid catabolic gene expression through NF-*κ*B. To test this hypothesis, HL-7702 cells were treated with 100 nM rapamycin (a specific inhibitor of mTORC1) for 8 hours, and the level of NF-*κ*B activation; the relative abundance of *AST*, *GDH*, *GAD*, and *ODC* mRNA; and the corresponding levels of intracellular enzymes were determined. The results showed that the inhibition of mTORC1 signal pathway by rapamycin reduces the phosphorylation level of IKK*α*, thus reducing the phosphorylation level of NF-*κ*B and attenuated degradation of I*κ*B*α* (Figures 1(a) and 1(b)) (*p* < 0.05), and the mRNA levels of *AST*, *GDH*, *GAD*, and *ODC* and the corresponding intracellular enzyme levels were significantly decreased by rapamycin treatment (Figures 1(c) and 1(d)) (*p* < 0.01). These results suggested that mTORC1 and NF-*κ*B are associated with the expression of these catabolic genes.

**Figure 2:**
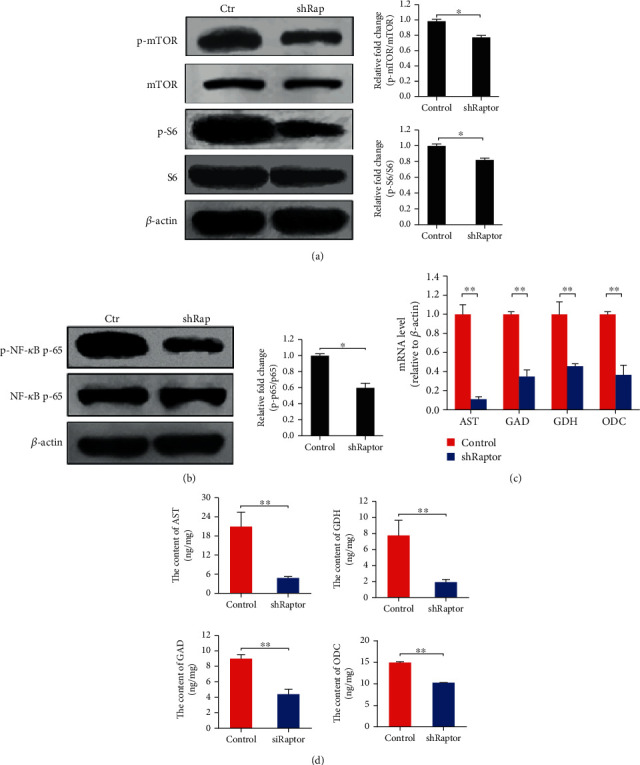
*Raptor* silencing impairs mTORC1 signaling and NF-*κ*B activation and decreases the expression of *AST*, *GDH*, *GAD*, and *ODC* in hepatocytes. (a) The phosphorylation of mTOR and S6 was reduced by *Raptor* silencing. (b) The phosphorylation of NF-*κ*B was reduced by *Raptor* silencing. (c) The relative mRNA levels of *AST*, *GDH*, *GAD*, and *ODC* were decreased by *Raptor* silencing. (d) The intracellular contents of AST, GDH, GAD, and ODC were decreased by *Raptor* silencing. Protein bands were quantified using Gel-Pro Analyzer 4.0 (Media Cybernetics, Inc., Rockville, MD, USA) (the values are presented as the means ± SD, *n* = 3, ^∗^*p* < 0.05, ^∗∗^*p* < 0.01).

To further evaluate the effects of inactivated mTORC1 on *AST*, *GDH*, *GAD*, and *ODC* expression, mTORC1 activation was reduced by knocking down *Raptor*, a critical component of mTORC1, using a *Raptor*-targeting shRNA (Figure S1), in HL-7702 cells, followed by the evaluation of S6 and NF-*κ*B phosphorylation. The phosphorylation of all target proteins was inhibited by *Raptor* silencing (Figures 2(a) and 2(b)). The levels of metabolic gene expression and intracellular enzymes were also measured, and the observed gene expression pattern was similar to that observed in rapamycin-treated cells (Figures 2(c) and 2(d)) (*p* < 0.01). These results further demonstrated that mTORC1 and NF-*κ*B are involved in catabolic gene expression.

### 3.2. Enhanced mTORC1 Activation Upregulates NF-*κ*B Activation and Amino Acid Catabolic Gene Expression in Hepatocytes

**Figure 3:**
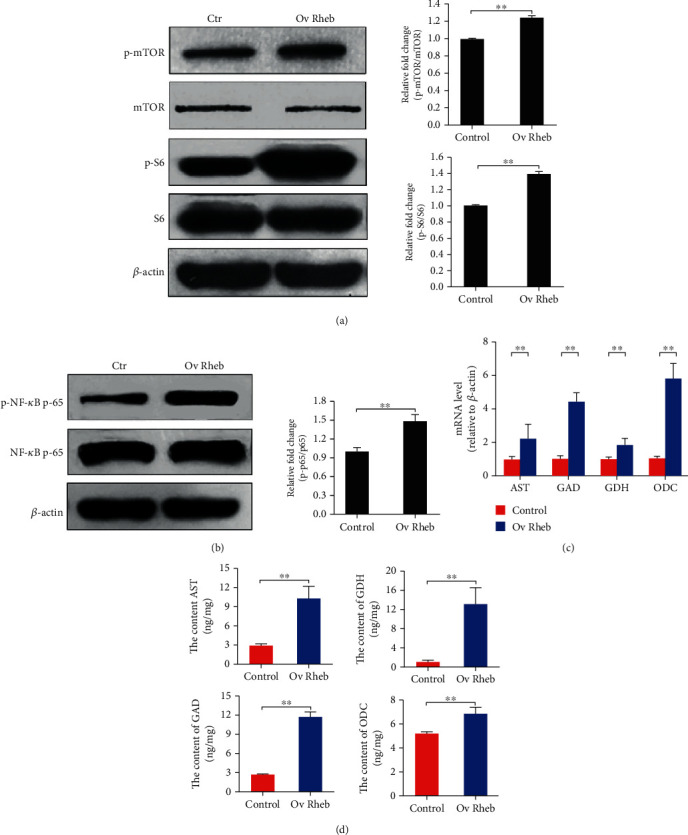
*Rheb* overexpression enhances mTORC1 signaling and NF-*κ*B activation and increases the expression of *AST*, *GDH*, *GAD*, and *ODC* in hepatocytes. (a) The phosphorylation of mTOR and S6 was promoted by *Rheb* overexpression. (b) The phosphorylation of NF-*κ*B was promoted by *Rheb* overexpression. (c) The relative mRNA levels of *AST*, *GDH*, *GAD*, and *ODC* were increased by *Rheb* overexpression. (d) The intracellular contents of AST, GDH, GAD, and ODC were increased by the overexpression of *Rheb.* Protein bands were quantified using Gel-Pro Analyzer 4.0 (Media Cybernetics, Inc., Rockville, MD, USA) (the values represent the means ± SD, *n* = 3, ^∗^*p* < 0.05, ^∗∗^*p* < 0.01).

To complement the results showing that *Raptor* silencing decreased mTORC1 activation and the expression of *AST*, *GDH*, *GAD*, and *ODC*, we cloned and overexpressed *Rheb*, an upstream positive effector of mTORC1, in HL-7702 cells, to enhance mTORC1 activation (Figure S2). We also measured NF-*κ*B activation; *AST*, *GDH*, *GAD*, and *ODC* expression; and the concentrations of the corresponding enzymes. *Rheb* overexpression upregulated mTORC1 signaling (Figure 3(a)) and NF-*κ*B phosphorylation (Figure 3(b)), and the expression levels of the examined catabolic genes were enhanced at both the mRNA (Figure 3(c)) (*p* < 0.01) and protein levels (Figure 3(d)) (*p* < 0.01). These results indicated that the expression levels of these catabolic genes and NF-*κ*B activation were increased by the enhanced activation of mTORC1.

### 3.3. NF-*κ*B Directs the Expression of AST, GDH, GAD, and ODC

**Figure 4:**
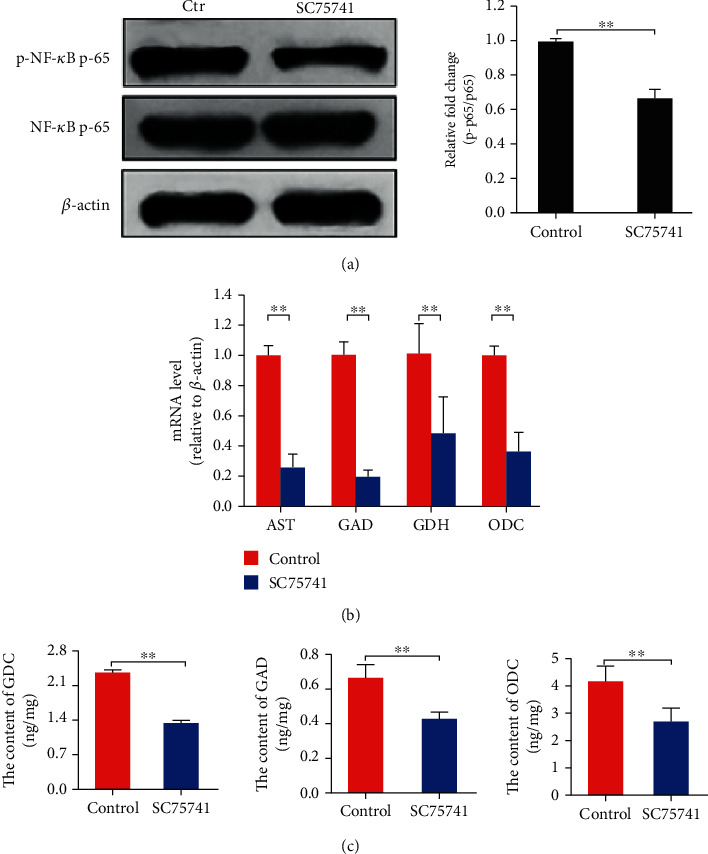
SC75741 inhibits NF-*κ*B activation and downregulates the expression levels of *AST*, *GDH*, *GAD*, and *ODC* in hepatocytes. (a) The phosphorylation of NF-*κ*B was inhibited by SC75741 treatment. (b) The relative mRNA levels of *AST*, *GDH*, *GAD*, and *ODC* were decreased by SC75741 treatment. (c) The intracellular contents of GDH, GAD, and ODC were decreased by SC75741 treatment. Protein bands were quantified using Gel-Pro Analyzer 4.0 (Media Cybernetics, Inc., Rockville, MD, USA) (the values represent the means ± SD, *n* = 3, ^∗^*p* < 0.05, ^∗∗^*p* < 0.01).

Based on the results showing that NF-*κ*B was associated with the expression of amino acid catabolic genes, we hypothesized that NF-*κ*B directs the expression of *AST*, *GDH*, *GAD*, and *ODC* in hepatocytes. To conform the hypothesis, we first performed a bioinformatics analysis using the UCSC Genome Browser and the PROMO database, and as a result, the putative transcription factor binding sites (TFBS) of the NF-*κ*B was found in the promoter sequence of the *AST* (Figure S3(a)), *GDH* (Figure S3(b)), *GAD* (Figure S3(c)), and *ODC* (Figure S3(d)), respectively. Further, to verify the effects of NF-*κ*B on the expression of *AST*, *GDH*, *GAD*, and *ODC*, we used SC75741, a specific inhibitor of NF-*κ*B, to inhibit NF-*κ*B activation in HL-7702 cells and then measured the levels of metabolic gene expression and intracellular enzymes. The results showed that NF-*κ*B activation was inhibited by the SC75741 (Figure 4(a)), and the mRNA levels of *AST*, *GDH*, *GAD*, and *ODC* and the corresponding intracellular enzyme levels were significantly decreased following SC75741 treatment (Figures 4(b) and 4(c)), respectively (*p* < 0.01), suggesting that NF-*κ*B may direct the expression of *AST*, *GDH*, *GAD*, and *ODC*.

**Figure 5:**
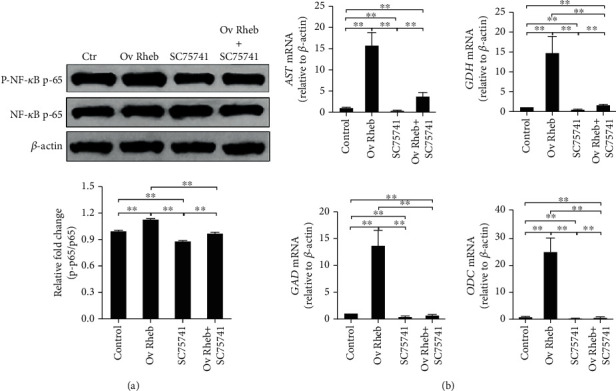
SC75741 inhibits amino acid catabolic gene expression in *Rheb*-overexpressing hepatocytes. *Rheb*-overexpressing hepatocytes were treated with 10 *μ*M SC75741 for 12 h, and then, NF-*κ*B phosphorylation and the expression levels of amino acid catabolic genes were determined. (a) NF-*κ*B activation was enhanced by *Rheb* overexpression and inhibited by SC75741 treatment. (b) The expression levels of amino acid catabolic genes were upregulated by *Rheb* overexpression and inhibited by SC75741 treatment. Protein bands were quantified using Gel-Pro Analyzer 4.0 (Media Cybernetics, Inc., Rockville, MD, USA) (the values represent the means ± SD, *n* = 3, ^∗^*p* < 0.05, ^∗∗^*p* < 0.01).

To further verify that mTORC1 regulates the expression of *AST*, *GDH*, *GAD*, and *ODC* through NF-*κ*B, *Rheb*-overexpressing HL-7702 cells were treated with 10 *μ*M SC75741 for 12 h, after which NF-*κ*B phosphorylation and the expression levels of the amino acid catabolic genes were evaluated. The results showed that NF-*κ*B activation was enhanced by *Rheb* overexpression and inhibited by SC75741 (Figure 5(a)), and the gene expression pattern was similar to pattern observed for NF-*κ*B phosphorylation (Figure 5(b)). These results indicated that mTORC1 likely controls the expression of these catabolic genes via NF-*κ*B in hepatocytes.

### 3.4. Glutamate and Ornithine Promote AST and ODC Expression, via the Activation of mTORC1 and NF-*κ*B in Hepatocytes

**Figure 6:**
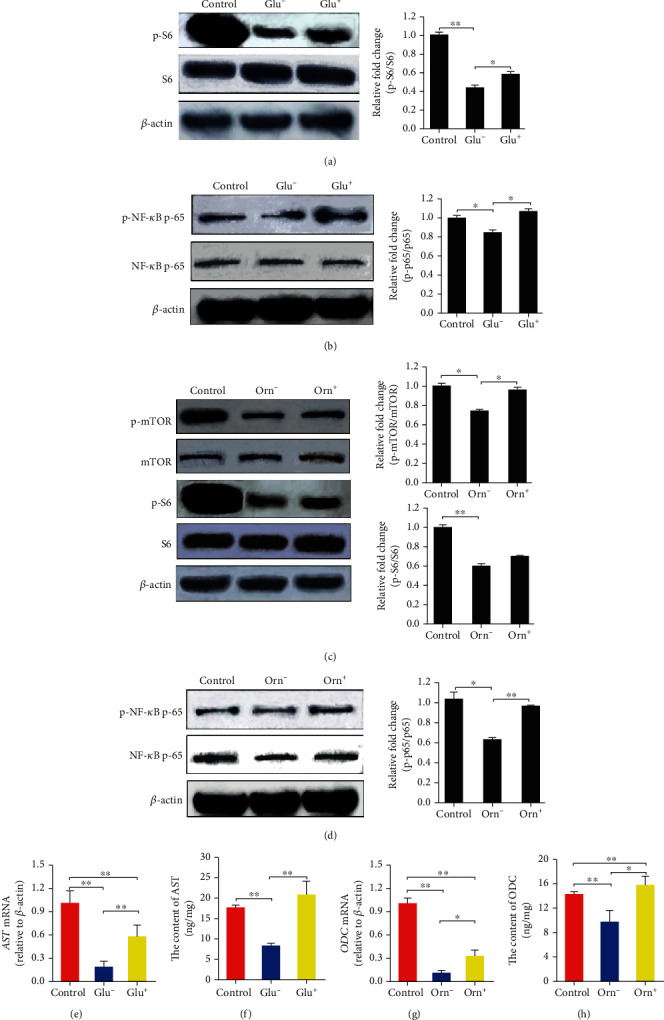
Glutamate and ornithine stimulate the activation of mTORC1 and NF-*κ*B signaling and promote the expression of *AST* and *ODC* in hepatocytes. (a) The activation of mTORC1 signaling was promoted by glutamate treatment. (b) The activation of the transcription factor NF-*κ*B was promoted by glutamate treatment. (c) The activation of mTORC1 signaling was promoted by ornithine treatment. (d) The activation of NF-*κ*B was promoted by ornithine treatment. (e) The relative abundance of *AST* mRNA was upregulated by glutamate treatment. (f) The intracellular content of AST was upregulated by glutamate. (g) The relative abundance of *ODC* mRNA was upregulated by ornithine treatment. (h) The intracellular content of ODC was upregulated by ornithine treatment. Protein bands were quantified using Gel-Pro Analyzer 4.0 (Media Cybernetics, Inc., Rockville, MD, USA) (the values represent the means ± SD, *n* = 3, ^∗^*p* < 0.05, ^∗∗^*p* < 0.01).

To confirm that mTORC1 regulates the expression of amino acid catabolic genes via NF-*κ*B, in response to extracellular amino acids, we used both a proteinogenic amino acid, glutamate, and a nonproteinogenic amino acid, ornithine, to treated serum- and amino acid-starved HL-7702 cells and then examined the relationship between mTORC1 activation and catabolic gene expression. The reversible transamination between glutamate and *α*-ketoglutaric acid can be catalyzed by AST, to produce oxaloacetate and aspartic acid, whereas the decarboxylation of ornithine into putrescine can be catalyzed by ODC; thus, we first examined the effects of glutamate and ornithine treatment on the activation of mTORC1 and NF-*κ*B signaling. Either glutamate or ornithine was added to starve HL-7702 cells, and the phosphorylation of S6, which is known to be phosphorylated in an mTORC1-dependent manner, was assessed. The phosphorylation of NF-*κ*B was also measured. The results showed that glutamate treatment significantly increased the phosphorylation of S6 and NF-*κ*B p65 (Figures 6(a) and 6(b)) compared with the starved, untreated group. Ornithine treatment increased the phosphorylation of mTOR and NF-*κ*B (Figures 6(c) and 6(d)). As a result, both the mRNA and protein expression levels of *AST* and *ODC* were increased by glutamate and ornithine treatment, respectively (Figures 6(e)–6(h)) (*p* < 0.05). These results suggested that mTORC1 signaling is responsible for the glutamate- and ornithine-induced upregulation of *AST* and *ODC* and that NF-*κ*B is likely associated with the expression of these genes in hepatocytes.

### 3.5. Rapamycin Inhibits the Utilization of Glutamate and Ornithine in Hepatocytes

**Figure 7:**
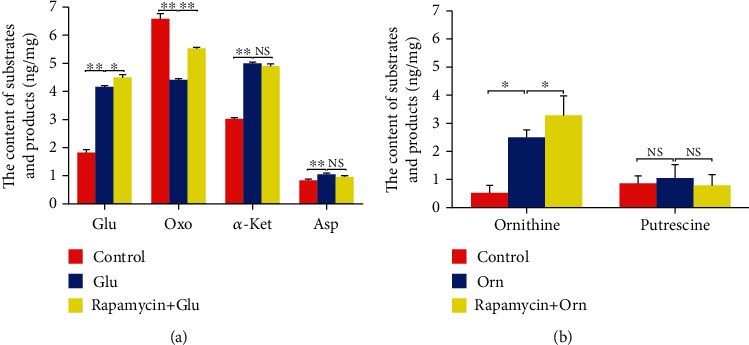
Rapamycin inhibits the transamination of glutamate and the decarboxylation of ornithine in hepatocytes. (a) Rapamycin inhibits the transamination between glutamate and *α*-ketoglutaric acid, to produce oxaloacetate and aspartic acid in cells. The contents of intracellular glutamate, oxaloacetate, *α*-ketoglutaric acid, and aspartic acid were measured by ELISA. The intracellular glutamate content in the group treated with glutamate was higher than that in the control group, and the glutamate level in the glutamate with the rapamycin group was higher than that in the glutamate alone group, indicating that rapamycin reduced the utilization of glutamate. The intracellular levels of *α*-ketoglutaric acid increased due to the activity of AST, which was attenuated by rapamycin. Treatment (the values represent the means ± SD, *n* = 3, ^∗^*p* < 0.05, ^∗∗^*p* < 0.01). (b) Rapamycin inhibits the ornithine decarboxylation necessary to produce putrescine, in cells. The contents of intracellular ornithine and putrescine were measured by HPLC. The intracellular ornithine content in the ornithine-treated group was higher than that in the control, and the ornithine group treated with both ornithine and rapamycin contained higher ornithine levels than those treated with ornithine alone, meaning that rapamycin reduced the utilization of ornithine (the values represent the means ± SD, *n* = 2, ^∗^*p* < 0.05).

To determine whether mTORC1 regulates the conversion between the substrate and the product during amino acid catabolism, the inhibitory effects of rapamycin on glutamate or ornithine catabolism were examined in hepatocytes. HL-7702 cells were starved and then divided into 3 groups: control, glutamate, and glutamate with rapamycin. The concentrations of intracellular glutamate, oxaloacetate, *α*-ketoglutaric acid, and aspartic acid were measured by ELISA. As shown in Figure 7(a), the intracellular glutamate concentration in the glutamate group was significantly higher than that in the control group (*p* < 0.01). The intracellular glutamate concentration of the glutamate with rapamycin group was significantly higher than that in the glutamate group (*p* < 0.05). These results indicated that rapamycin likely reduced the utilization of glutamate.

Furthermore, the intracellular oxaloacetate concentration of the glutamate group was significantly lower than that in the control group (*p* < 0.01). The intracellular oxaloacetate concentration of the glutamate with rapamycin group was significantly higher than that in the glutamate group (*p* < 0.01). These data indicated that the utilization of oxaloacetate was likely blocked by rapamycin. The intracellular concentrations of *α*-ketoglutaric acid and aspartic acid were higher in the glutamate group compared with those in the control group (*p* < 0.01). These results suggested that rapamycin likely prevented the accumulation of *α*-ketoglutaric acid.

Ornithine is converted into a polyamine by ODC. To characterize the inhibitory effects of rapamycin on ornithine catabolism, HL-7702 cells were starved and then divided into 3 groups: control, ornithine, and ornithine with rapamycin. The concentrations of intracellular ornithine and putrescine were measured by HPLC. The intracellular ornithine concentration in the ornithine group was significantly higher than that in the control group (Figure 7(b)) (*p* < 0.05), and the ornithine content of the ornithine with the rapamycin group was significantly higher than that in the ornithine group (*p* < 0.05). These data indicated that rapamycin likely blocked ornithine utilization.

## 4. Discussion

Amino acid catabolism supplies energy and macromolecule synthesis precursor to support cellular functions, and key enzymes play important roles in this process. In this study, we demonstrated that mTORC1 regulates the expression of amino acid catabolic genes in hepatocytes. Two recent reports showed that plasma AST levels were regulated by mTORC1 in rats [24, 37]. GDH activity has been associated with mTORC1 activity in ovarian cancer cells [25], and prolactin induces the ODC expression, via mTOR signaling, in mink uterine epithelial cells [38]; however, a relationship between *GAD* and mTOR signaling has not been reported. In our study, the expression of *AST*, *GDH*, *GAD*, and *ODC* was inhibited by both rapamycin treatment and *Raptor* silencing. Additionally, the intracellular protein levels of AST, GDH, GAD, and ODC were regulated by mTORC1. Further, the conversion of glutamate to *α*-ketoglutarate and the utilization of ornithine were both controlled by mTORC1.

The NF-*κ*B signaling pathway plays a well-known and significant role during inflammation. In recent years, NF-*κ*B has been thought to play a role in cell metabolism [27–31]. Feist et al. [30] reported that cooperative NF-*κ*B/STAT3 signaling is associated with lymphoma metabolic reprogramming and aspartate transaminase (GOT2) gene expression. In our previous study, we found that mTORC1 regulates peptidoglycan-induced inflammation, via NF-*κ*B, in murine macrophages [32]. In the present study, we focused on the regulation of NF-*κ*B and effects on the amino acid catabolic gene expression. We found that the expression of *AST*, *GDH*, *GAD*, and *ODC* was regulated by the mTORC1/NF-*κ*B axis. Thus, we concluded that mTORC1 regulates the expression of amino acid catabolic genes via NF-*κ*B. However, whether similar alterations in the transcription of amino acid catabolizing enzymes occur under other conditions known to stimulate NF-*κ*B activation remains unknown. This study has several limitations. First, although we have studied the regulatory functions of mTORC1 on the amino acid gene expression via NF-*κ*B *in vitro*, these findings must be verified *in vivo*. Second, we observed that NF-*κ*B mediates the signals between mTORC1 and the transcriptional control of amino acid catabolic genes; however, we did not examine whether similar alterations in the catabolic gene expression occurs under other conditions during which NF-*κ*B is stimulated. Third, glutamate and ornithine are not essential amino acids and can be synthesized by cells. Therefore, the levels of glutamate and ornithine in cells are determined by more complex mechanisms than the mere addition of glutamate and ornithine to the culture medium or the catabolism of glutamate. Similarly, oxaloacetate, ornithine, and putrescine levels can also be affected by multiple sources and metabolic pathways in cells. However, the utilization of glutamate and ornithine in cells was significantly inhibited by rapamycin treatment.

Over the past 10 years, only a few research groups have examined the connection between mTORC1 and NF-*κ*B, and reports have been published discussing the involvement of mTOR/Raptor in the control of the NF-*κ*B target gene expression [39], the finding that mTORC1/NF-*κ*B signaling comediated tumor necrosis factor *α*-induced apoptosis [40] and the effects of branched-chain amino acids on NF-*κ*B p65 activation via mTORC1 and nuclear translocation [41]. In our previous study, we reported that mTORC1 mediates peptidoglycan or flagellin-induced inflammatory responses in macrophages [32, 36]. However, the mechanism through which mTORC1 regulates NF-*κ*B remains unclear. A recent report by Li et al. [34] showed that mTORC1 bound to and phosphorylated both IKK*α* and IKK*β*, which enhanced their kinase activities. IKK*α*/IKK*β* subsequently phosphorylated I*κ*B, inducing the release and activation of NF-*κ*B in rat pulmonary artery smooth muscle cells, which were derived from a rat model of hypoxia-induced pulmonary hypertension.

**Figure 8:**
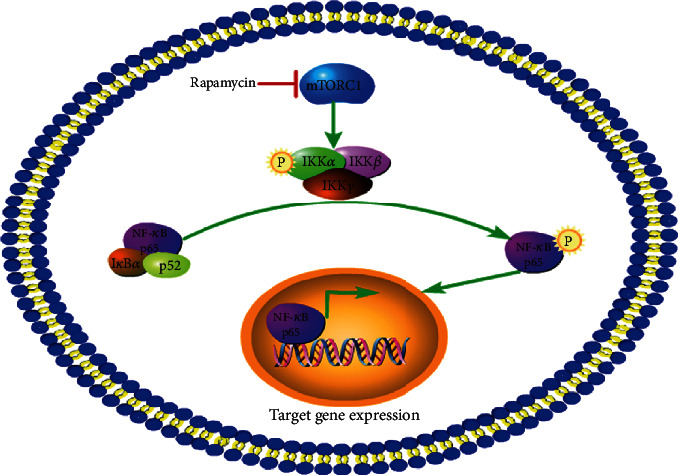
Diagram of the interaction of mTOR and NF-*κ*B in human hepatocytes.

In conclusion, this study examined the function of mTORC1 during amino acid catabolism in hepatocytes, demonstrating that mTORC1 regulates the expression of amino acid metabolic genes, including *AST*, *GDH*, *GAD*, and *ODC*, through NF-*κ*B (Figure 8). Furthermore, mTORC1 signaling is responsible for the response to glutamate and ornithine, increasing the expression of *AST* and *ODC* through NF-*κ*B, and controlling the utilization of glutamate and ornithine in hepatocytes. This study provides a possible mechanism for amino acid catabolism in human hepatocytes.

## Data Availability

The data used to support the findings of this study are included within the article.

## Supplementary Materials

Supplementary materials Table S1: the primers for amplifying cDNA sequences of target genes. Figure S1: silencing *Raptor* with shRNA in hepatocytes. (a) pRNAT-U6.1/Neo-Raptor-shRNA-transfected cells, expressing EGFP. (b) *Raptor* mRNA level was decreased in cells transfected with pRNAT-U6.1/Neo-Raptor-shRNA. (c) Raptor protein level was decreased in cells transfected with pRNAT-U6.1/Neo-Raptor-shRNA. Protein bands were quantified using Gel-Pro Analyzer 4.0 (Media Cybernetics, Inc., Rockville, MD, USA) (the values represent the means ± SD, *n* = 3, ^∗∗^*p* < 0.01). Figure S2: overexpression of *Rheb* in hepatocytes. (a) pIRES2-EGFP-Rheb-transfected cells expressing EGFP. (b) *Rheb* mRNA level was increased in cells transfected with pIRES2-EGFP-Rheb. (c) Rheb protein level was increased in cells transfected with pIRES2-EGFP-Rheb. Protein bands were quantified using Gel-Pro Analyzer 4.0 (Media Cybernetics, Inc., Rockville, MD, USA) (the values represent the means ± SD, *n* = 3, ^∗∗^*p* < 0.01). Figure S3: the putative transcription factor binding sites (TFBS) of NF-*κ*B in the promoter sequence of *AST*, *GDH*, *GAD*, and *ODC*. (a) The NF-*κ*B TFBS in the promoter sequence of *AST*. (b) The NF-*κ*B TFBS in the promoter sequence of *GDH*. (c) The NF-*κ*B TFBS in the promoter sequence of *GAD*. (d) The NF-*κ*B TFBS in the promoter sequence of *ODC*. Figure S4: the putative transcription factor binding motifs (TFBM) of NF-*κ*B in the promoter sequence of AST, GDH, GAD, and ODC (red boxes represent motif). (a) The NF-*κ*B TFBM in the promoter sequence of AST ((-160, -1034, -1143, -1335, -1562) *p* < 0.001). (b) The NF-*κ*B TFBM in the promoter sequence of GDH ((-1865, -1395, -423, -367) *p* < 0.001). (c) The NF-*κ*B TFBM in the promoter sequence of GAD ((-1952, -1753, -1752, -1554, -1376, -969, -936, -885, -442, -80, -79) *p* < 0.001). (d) The NF-*κ*B TFBM in the promoter sequence of ODC ((-1158, -1122, -1038, -966, -900, -744, -618, -460) *p* < 0.001). (e) The TFBM of NF-*κ*B of Jaspar Database.
