# Supplementary material for: Inhibition of the mTORC1/NF-κB Axis Alters Amino Acid Metabolism in Human Hepatocytes
Source: Biomed Res Int. 2021 Jan 18;2021:8621464. doi: 10.1155/2021/8621464 (PMC7843190; doi:10.1155/2021/8621464)
Supplement: Supplementary materials — Table S1: the primers for amplifying cDNA sequences of target genes. Figure S1: silencing Raptor with shRNA in hepatocytes. (a) pRNAT-U6.1/Neo-Raptor-shRNA-transfected cells, expressing EGFP. (b) Raptor mRNA level was decreased in cells transfected with pRNAT-U6.1/Neo-Raptor-shRNA. (c) Raptor protein level was decreased in cells transfected with pRNAT-U6.1/Neo-Raptor-shRNA. Protein bands were quantified using Gel-Pro Analyzer 4.0 (Media Cybernetics, Inc., Rockville, MD, USA) (the values represent the means ± SD, n = 3, ∗∗p < 0.01). Figure S2: overexpression of Rheb in hepatocytes. (a) pIRES2-EGFP-Rheb-transfected cells expressing EGFP. (b) Rheb mRNA level was increased in cells transfected with pIRES2-EGFP-Rheb. (c) Rheb protein level was increased in cells transfected with pIRES2-EGFP-Rheb. Protein bands were quantified using Gel-Pro Analyzer 4.0 (Media Cybernetics, Inc., Rockville, MD, USA) (the values represent the means ± SD, n = 3, ∗∗p < 0.01). Figure S3: the putative transcription factor binding sites (TFBS) of NF-κB in the promoter sequence of AST, GDH, GAD, and ODC. (a) The NF-κB TFBS in the promoter sequence of AST. (b) The NF-κB TFBS in the promoter sequence of GDH. (c) The NF-κB TFBS in the promoter sequence of GAD. (d) The NF-κB TFBS in the promoter sequence of ODC. Figure S4: the putative transcription factor binding motifs (TFBM) of NF-κB in the promoter sequence of AST, GDH, GAD, and ODC (red boxes represent motif). (a) The NF-κB TFBM in the promoter sequence of AST ((-160, -1034, -1143, -1335, -1562) p < 0.001). (b) The NF-κB TFBM in the promoter sequence of GDH ((-1865, -1395, -423, -367) p < 0.001). (c) The NF-κB TFBM in the promoter sequence of GAD ((-1952, -1753, -1752, -1554, -1376, -969, -936, -885, -442, -80, -79) p < 0.001). (d) The NF-κB TFBM in the promoter sequence of ODC ((-1158, -1122, -1038, -966, -900, -744, -618, -460) p < 0.001). (e) The TFBM of NF-κB of Jaspar Database. [file 8621464.f1.docx]

**Supplementary File 1**

**1. The primers for amplifying cDNA sequences of target genes in Table S1**

Total RNA was isolated using RNAzol (9109, TaKaRa Co. Ltd., Dalian, China) from cells, respectively, and was reverse-transcribed with an oligo (dT)_12–18_ primer using the EasyScript® One-Step gDNA Removal and cDNA Synthesis SuperMix Kit (AE311, TransGen Biotech Co. Ltd., Beijing, China). The transcription of *AST,* *GDH*, *GAD*, *ODC*, *Rheb* and *Raptor* was detected by RT-qPCR, and cDNA sequences were amplified with the primers in Table S1.

**Table S1 the primers for amplifying cDNA sequences of target genes**

| Gene Name Gene ID Forward primer (5'-3') Reverse primer (5'-3') | |  |  |
| --- | --- | --- | --- |
| \| *AST* \| 26503 \| CCTTCGTATGCTGGTATCCT \| TTGTACTTCACCTTTGGCG \| \| --- \| --- \| --- \| --- \| \| *GDH*  *GAD* \| 2746 \| GCTGGAGGAGTGACAGTATCTT \| TGGAACTCTGCCGTGGGTA \| \| 2571 \| GGCAATCCTCCAAGAACCT \| TGATGAAAGTCCAGCACCT \| \| *ODC* \| 4953 \| TGTGGGTGATTGGATGCTC \| GGCTGCTCTGTGGCGTTT \| \| *Rheb* \| 6009 \| GTTGGTTGGGAATAAGAAAGAC \| CACATCACCGAGCATGAAGACT \| \| *Raptor* \| 57521 \| GAGCAGGTGACTAAGGAAGAC \| CAGGTGCCGAGAGTGAAG \| |  | |  |
|  |  |  |  |
|  |  | | |

**2. *Raptor* knockdown in hepatocytes**

According to the sequence of human *Raptor* gene (GenBank: AY090663.1), a short hairpin RNA (shRNA)-based *Raptor* RNA-silencing construct (shRaptor), with the sequence 5′-aaGCTCTGCACGTCCTTACGTTTCAAGAGAACGTAAGGACGTGCAGAGCtt-3′, was designed and synthesized to construct the pRNAT-U6.1/Neo-shRaptor expression vector. The plasmid pRNAT-U6.1/Neo-shRaptor was transfected into HL-7702 cells, using Lipofectamine 2000 (Invitrogen, Carlsbad, New Mexico, USA), according to the manufacturer’s instructions. Transfectants were selected by culturing cells in the presence of G418 (Hyclone Laboratories, Inc. Logan, Utah, USA), for 48 hours, and were imaged using a ZEISS AX10 fluorescence microscope (Carl Zeiss Microscopy, Thornwood, NY, USA), before the cells were collected (Figure S1(a)). The expression of *Raptor* in transcription level and protein level was detected by RT-qPCR and western bolt, respectively. Compared with the control group, the transcription level of *Raptor* was downregulated in *Raptor* knockdown cells (Figure S1(b)), and the Raptor protein also was decreased (Figure S1(c)).


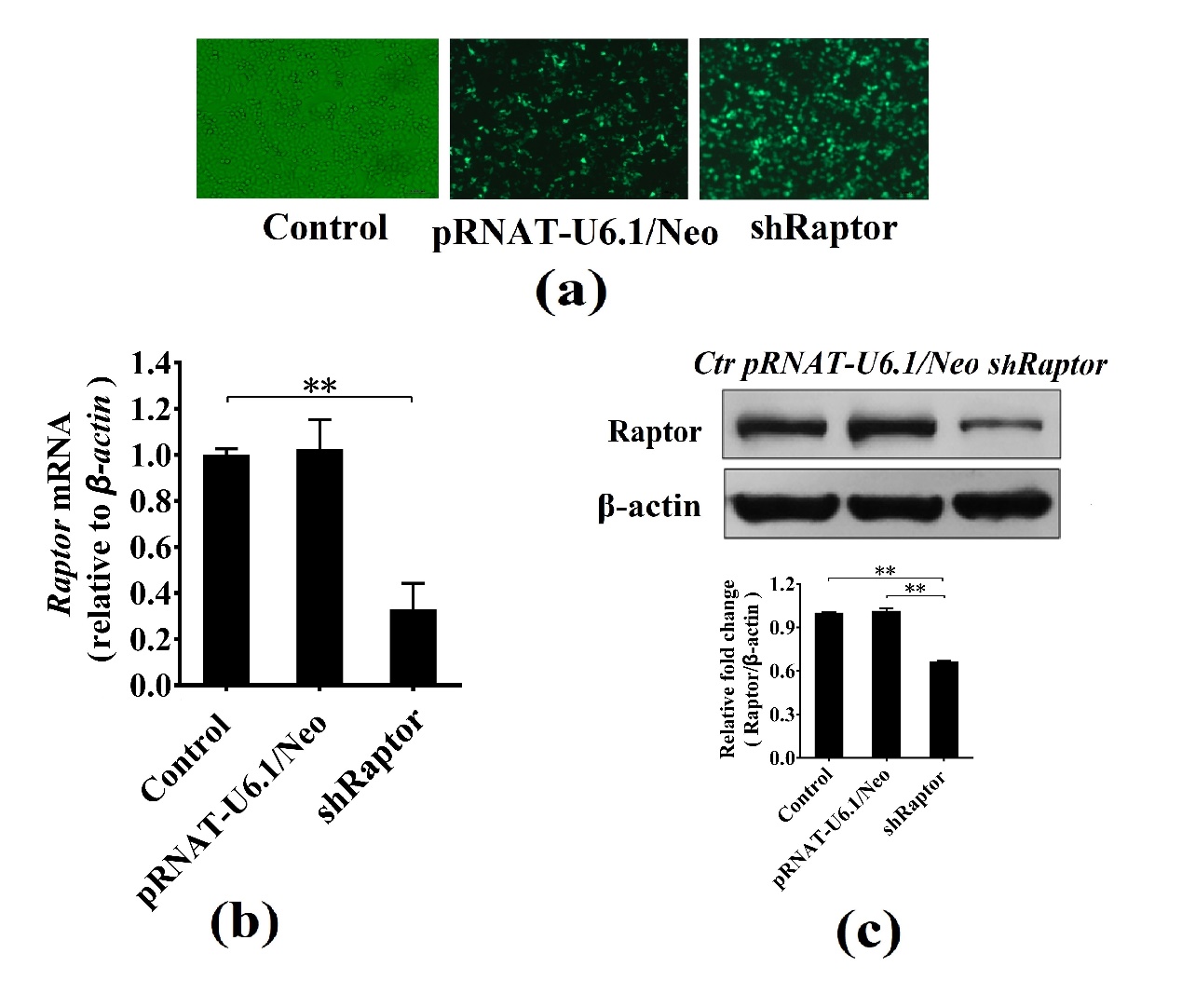


**FIGURE S1** Silencing *Raptor* with shRNA in hepatocytes

(a) pRNAT-U6.1/Neo-Raptor-shRNA-transfected cells, expressing EGFP.

(b) *Raptor* mRNA level was decreased in cells transfected with pRNAT-U6.1/Neo-Raptor-shRNA.

(c) Raptor protein level was decreased in cells transfected with pRNAT-U6.1/Neo-Raptor-shRNA. Protein bands were quantified using Gel-Pro Analyzer 4.0 (Media Cybernetics, Inc., Rockville, MD, USA). (The values represent the means±SD, n = 3, ** *p* < 0.01).

3. **Overexpression of *Rheb* in hepatocytes**

*Rheb* cDNA was amplified, using the forward primer 5'-GTTGGTTGGGAATAAGAAAGAC-3' and the reverse primer 5'-CACATCACCGAGCATGAAGACT-3', which were based on the human *Rheb* sequence (GenBank Accession number NM_005614). The *Rheb* PCR fragment was inserted into the pIRES2-EGFP vector (Clontech Laboratories, Inc., Mountain View, CA, USA) to construct the pIRES2-EGFP-Rheb expression vector. The plasmid pIRES2-EGFP-Rheb was transfected into HL-7702 cells, using Lipofectamine 2000 (Invitrogen, Carlsbad, New Mexico, USA), according to the manufacturer’s instructions. Transfectants were selected by culturing cells in the presence of G418 (Hyclone Laboratories, Inc. Logan, Utah, USA), for 48 hours, and were imaged using a ZEISS AX10 fluorescence microscope (Carl Zeiss Microscopy, Thornwood, NY, USA), before the cells were collected (Figure S2(a)). Compared with the control group, the transcription level of *Rheb* was upregulated in the *Rheb* overexpression cells (Figure S2(b)), and the Rheb protein also was increased (Figure S2(c)).


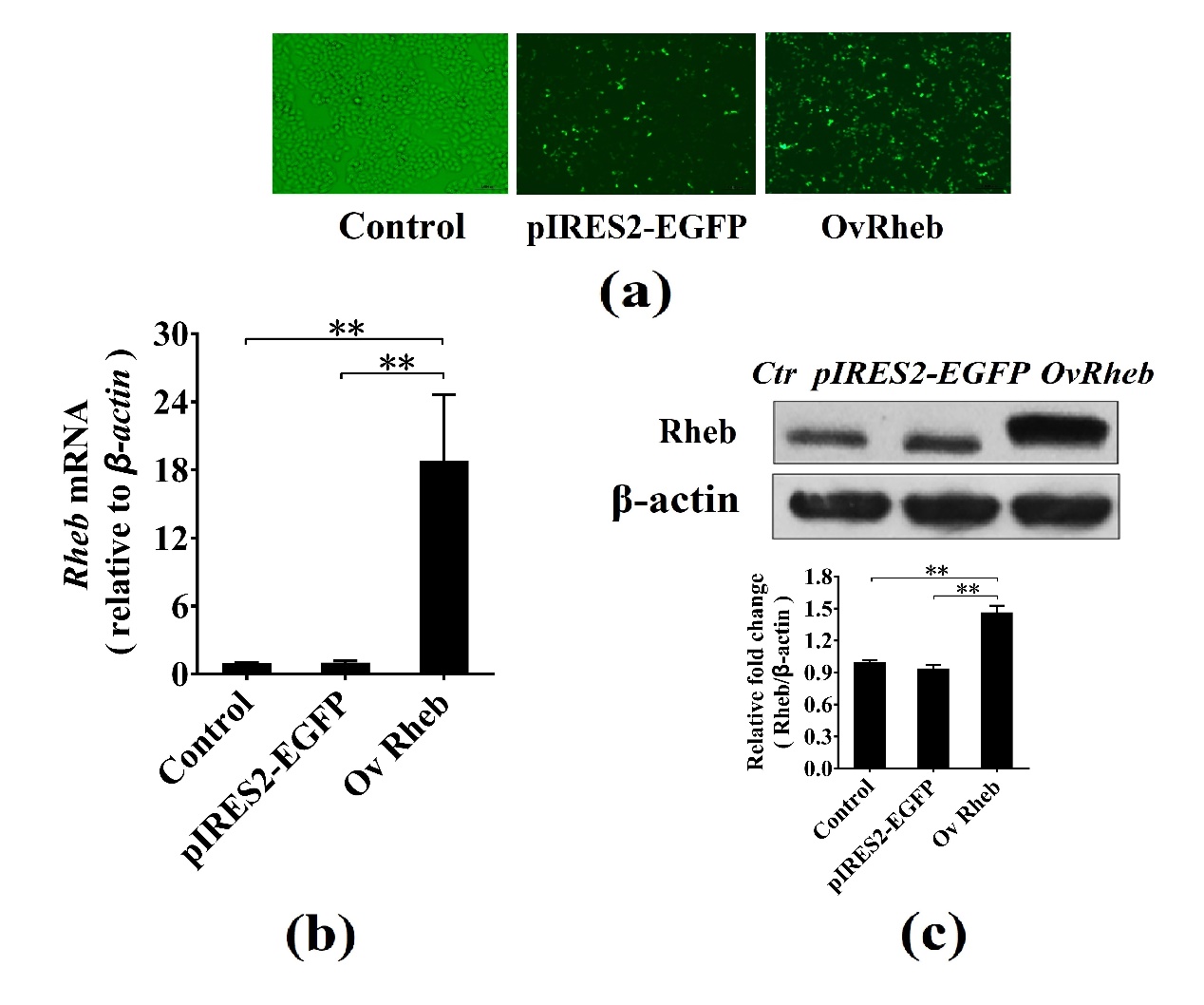


**FIGURE S2** Overexpression of *Rheb* in hepatocytes

(a) pIRES2-EGFP-Rheb-transfected cells expressing EGFP.

(b) *Rheb* mRNA level was increased in cells transfected with pIRES2-EGFP-Rheb.

(c) Rheb protein level was increased in cells transfected with pIRES2-EGFP-Rheb. Protein bands were quantified using Gel-Pro Analyzer 4.0 (Media Cybernetics, Inc., Rockville, MD, USA). (The values represent the means±SD, n = 3, ** *p* < 0.01).

**4. Prediction of putative transcription factor binding sites (TFBS) and the transcription factor binding motifs (TFBM) of the transcription factor NF-κB in the promoter sequence of the *AST*, *GDH*, *GAD* and *ODC***

The TFBS of NF-κB was analysed in the promoter sequence of the *AST*, *GDH*, *GAD* and *ODC* by bioinformatics analysis using the UCSC Genome Browser (<http://genome.ucsc.edu/index.html>) and the PROMO database (<http://alggen.lsi.upc.es/cgi-bin/promo_v3/promo/promoinit.cgi?dirDB=TF_8.3>), and as a result, the TFBS of the NF-κB was found in the promoter sequence of the *AST* (Figure S3(a)), *GDH* (Figure S3(b)), *GAD* (Figure S3(c)) and *ODC* (Figure S3(d)), respectively. The UCSC Genome Browser was used to find the promoter sequences of the *AST*, *GDH*, *GAD* and *ODC*, and the PROMO database was used to identify the putative transcription factor binding sites (TFBS) of NF-κB in the promoter sequence of *AST*, *GDH*, *GAD* and *ODC*, respectively. The transcription factor NF-κB was predicted within a dissimilarity margin less or equal than 15%.


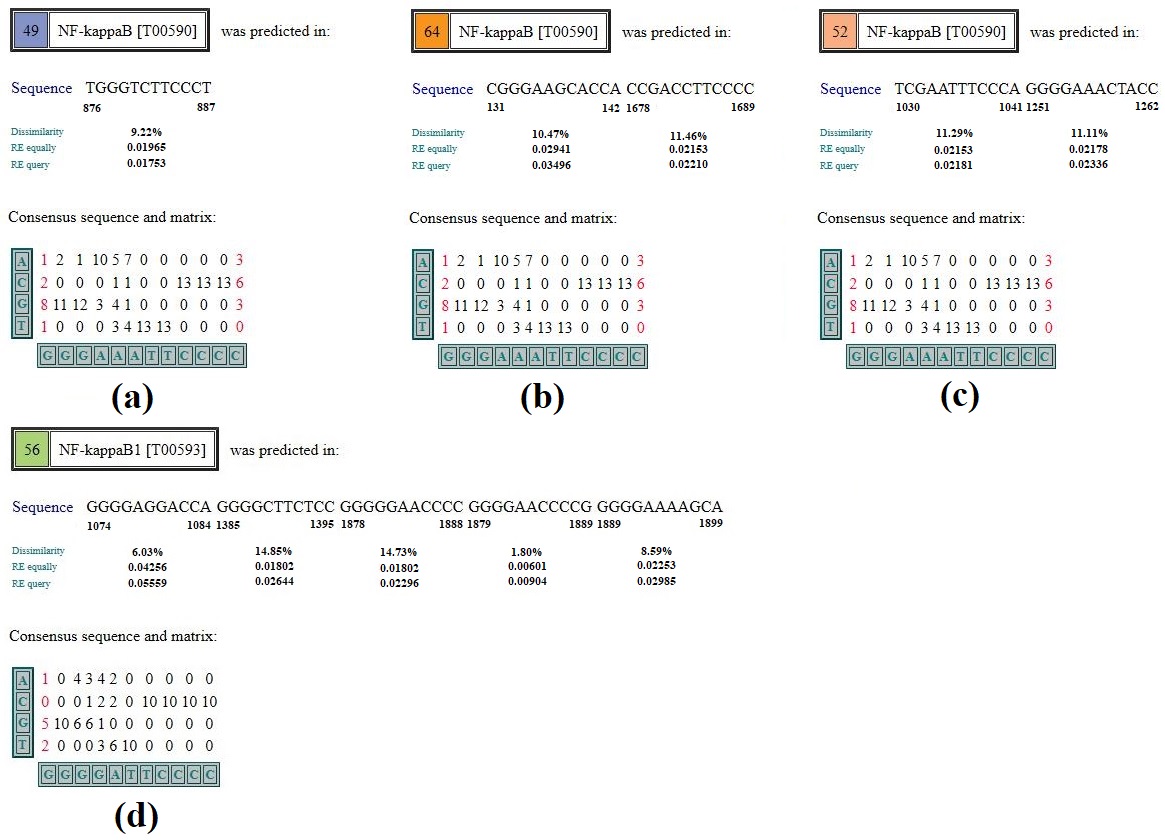


**FIGURE S3** The putative transcription factor binding sites (TFBS) of NF-κB in the promoter sequence of *AST*, *GDH*, *GAD* and *ODC*.

(a) The NF-κB TFBS in the promoter sequence of *AST*.

(b) The NF-κB TFBS in the promoter sequence of *GDH*.

(c) The NF-κB TFBS in the promoter sequence of *GAD*.

(d) The NF-κB TFBS in the promoter sequence of *ODC*.

The TFBM of NF-κB were predicted in the promoter sequence of the *AST*, *GDH*, *GAD* and *ODC* by bioinformatics analysis using the Eukaryotic Promoter Database (https://epd.epfl.ch//index.php) and the Jaspar Database ([http://jaspar.genereg.net/](http://alggen.lsi.upc.es/cgi-bin/promo_v3/promo/promoinit.cgi?dirDB=TF_8.3)). The promoter sequences were selected from -2000 to 100 bp relative to TSS and a cut-off, using Eukaryotic Promoter Database. The TFBM of the NF-κB were defined by Jaspar Database, respectively. And as a consequence, the motifs of the NF-κB was found in the promoter sequence of the *AST* (Figure S4(a)), *GDH* (Figure S4(b)), *GAD* (Figure S4(c)) and *ODC* (Figure S4(d)) (*p* < 0.001).


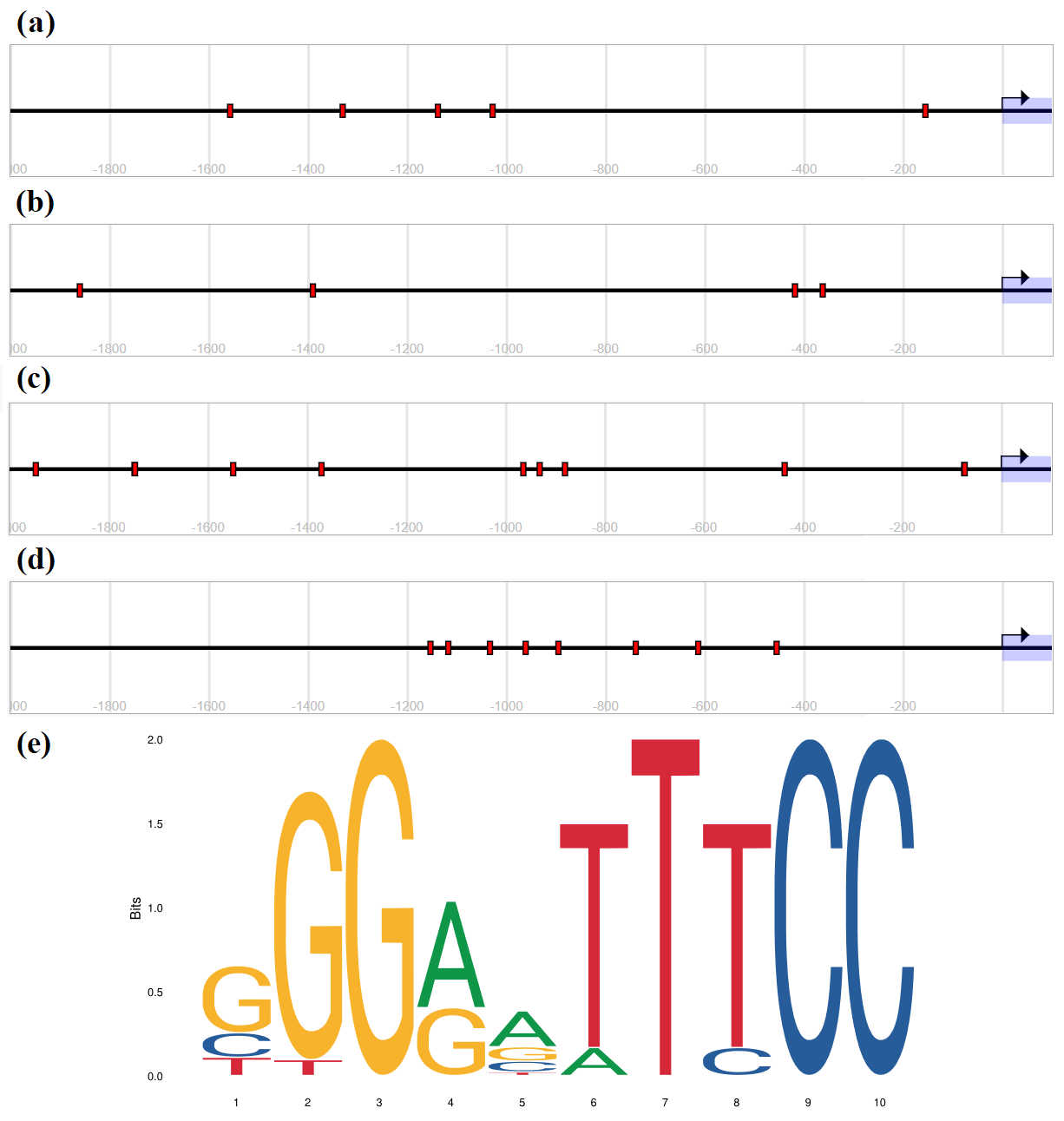


**FIGURE S4** The putative transcription factor binding motifs (TFBM) of NF-κB in the promoter sequence of *AST*, *GDH*, *GAD* and *ODC* (Red boxes represent motif).

(a) The NF-κB TFBM in the promoter sequence of *AST* ((-160, -1034, -1143, -1335, -1562) *p* < 0.001).

(b) The NF-κB TFBM in the promoter sequence of *GDH* ((-1865, -1395, -423, -367) *p* < 0.001).

(c) The NF-κB TFBM in the promoter sequence of *GAD* ((-1952, -1753, -1752, -1554, -1376, -969, -936, -885, -442, -80, -79) *p* < 0.001).

(d) The NF-κB TFBM in the promoter sequence of *ODC* ((-1158, -1122, -1038, -966, -900, -744, -618, -460) *p* < 0.001).

(e) The TFBM of NF-κB of Jaspar Database.
